# Women in epidemiology

**DOI:** 10.1017/ash.2022.351

**Published:** 2022-12-22

**Authors:** Judith A. Guzman-Cottrill

**Affiliations:** Division of Infectious Diseases, Department of Pediatrics, Oregon Health and Science University School of Medicine, Portland, Oregon

## Tell us about your career journey, specifically, your unique and successful integration of public health and hospital epidemiology

I finished my pediatric infectious diseases fellowship at Northwestern University Children’s Memorial Hospital (now Lurie Children’s Hospital) in 2004. After fellowship, I joined the Oregon Health and Science University (OHSU) faculty as a clinician educator. At the time, my passions were patient care and medical education. However, my new job also included the role of infection control medical director for the children’s hospital.

My fellowship training did not include significant exposure to hospital epidemiology or infection prevention and control (IPC). At the time, I honestly saw this role as a “small part” of my new job and not a career pathway. I thought the role would consist of quarterly committee meetings and not much more. I quickly realized that I had a lot to learn! I spent countless hours in the IPC office. I did chart reviews alongside the infection preventionists and learned all about the National Healthcare Safety Network (NHSN) from our program manager, Mary Post. I joined the Society for Healthcare Epidemiology of America (SHEA) and attended the SHEA/Centers for Disease Control and Prevention (CDC) Training Certificate Course in Healthcare Epidemiology. The training course was an excellent introduction; it provided the perfect foundation for my new role. This training occurred around the same time that the Institute for Healthcare Improvement (IHI) bundles created a novel, nationwide focus on healthcare-associated infection (HAI) prevention. It was an exciting time to be on the IPC team. While strategizing how to translate the adult-focused bundles to pediatric HAI prevention bundles, I met all the key leaders across the healthcare system. These leaders became colleagues that helped me with many “wins” during my time as the IPC pediatric medical director and hospital epidemiologist. My IPC colleague Dr. John Townes and I slowly grew our IPC program. Over the years, hospital epidemiology became my passion and career focus. I never looked back.

There is a saying: *All good things must come to an end*. In 2015, I ended up leaving the job that I loved. I found myself in a situation in which the hospital leadership and I had developed different guiding principles. It was a difficult career moment; I almost left academics completely. But after endless hours of discussion with colleagues and friends, I resigned from my IPC medical directorship. I stayed on part-time in the Department of Pediatrics as a clinician educator, and I am still an active faculty member at OHSU.

Around that same time, the Oregon Department of Health’s HAI program received federal funding to create an Ebola preparedness program. I was invited to serve as Medical Director of Ebola Preparedness for the entire state. It was an incredible opportunity to continue doing infection prevention in a completely new way. I decided to assume this role as an independent contractor.

I started my own infection prevention consulting business, Infection Prevention Consulting of Oregon. With the help of many nonmedical friends, I learned how to establish a limited liability corporation (LLC), how to negotiate my consulting contract, how to obtain appropriate liability insurance, and how to build a business website. Since 2015, 7 years ago, I have maintained a continuous consulting contract with my state’s HAI program. Beyond the Ebola preparedness work, my contract has led to an ever-changing variety of topics. I have assisted in several outbreaks, including measles. I recruited hospitals to begin reporting to the NHSN Antimicrobial Use and Resistance (AUR) module. In 2020, my public health work extended beyond healthcare facilities. I helped write state-level coronavirus disease 2019 (COVID-19) infection prevention guidance for both healthcare and school settings. I taught Oregon’s K–12 schools how to launch our COVID-19 antigen-testing program. During the initial COVID-19 vaccine rollout, I educated clinicians on the importance of reporting vaccine adverse events to the Vaccine Adverse Event Reporting System (VAERS). Currently, I am the medical director for Oregon Project Firstline, a CDC-led infection prevention educational initiative. After all of the COVID-19 work, it is nice to return to basic infection prevention education!

Working in public health has also exposed me to emergency responses through many different lenses. I am always learning. For example, when the most destructive wildfires in Oregon’s history encroached on the city of Portland in September 2020, I was involved in emergency meetings to quickly modify COVID-19 mitigation requirements to allow for fire-related evacuations. We wrote guidance for crowded shelters filled with evacuees; this was before COVID-19 vaccines were available. Instead of keeping city bus windows open to improve ventilation, we instructed the transit authority to close all the windows because of hazardous air quality. The overlap of 2 devastating emergencies that required opposing responses was dizzying.

## Tell us about the role of mentorship in shaping your career; describe a pivotal mentor relationship either within your institution or within SHEA that altered the trajectory of your career

While attending the SHEA/CDC training course (18 years ago), I distinctly remember an observation that left me uneasy: Only a handful of women were speakers, including Dr. Trish Perl. I also noticed that most speakers were white men from the east coast. I felt like a fish out of water—a Filipino female pediatrician from the west coast with a D.O. behind my name. I left the meeting wondering if SHEA represented me (personally and professionally).

So many women in SHEA have been mentors to me, both from afar and as colleagues. I have watched several women in epidemiology move up the ranks from afar. I remember Dr. Trish Perl’s lectures at the training course; she was so down to earth and really spoke her mind. I watched Dr. Perl’s eventual rise to the SHEA presidency. She remains a distant inspiration to me, and I finally told her this (in person) at the SHEA Spring 2022 meeting!

Another great mentor is Dr. Louise Dembry. We served on the SHEA Board of Trustees at the same time. During our time as board members, Louise repeatedly pulled me aside to deliver quick career advice. She taught me to be persistent and to share my ideas in the board room. Louise has remained a cheerleader for my career successes. We all need career cheerleaders!

Dr. Rekha Murthy is not only a mentor, but a colleague and friend. We became colleagues while serving on the SHEA Board of Trustees, and I have learned so much from Rekha. Her career success at Cedars-Sinai has been one of perseverance. She is incredibly calm, all the time. Rekha’s enthusiasm behind my decision to leave my hospital epidemiologist position and pave my own way as an independent consultant gave me the validation I needed. I am so grateful for her career support.

Finally, I must mention my husband Jerod, who is also my career cheerleader. He constantly reminds me that my expertise is incredibly valuable. He pushes me to continually raise expectations for myself and my consulting business. He’s also a physician, but we have very different careers. He tells everyone that my job is more important than his job!

## What were and are the biggest drivers of your success?

Early in my career, I worked with a chief medical officer who passed down “simple advice” from his own mentor: *Proceed until apprehended*. I am amazed by how frequently I follow this advice. If I am passionate about a new initiative or idea, I will do everything I can to see it come to fruition. I have not been apprehended yet!

For many years, my biggest motivator was proving that I was a capable leader. I had to prove to myself that I could be as successful as my peers who worked at larger academic centers with all the resources at their fingertips. I got involved with the SHEA Pediatric Leadership Council, which provided many volunteer opportunities. I worked hard to finish every task I signed up for.

In 2014, I was invited to serve as the Pediatric Infectious Disease Society (PIDS) liaison to the SHEA Board of Trustees. This was my first opportunity to prove myself as a leader at the national level, and I had 3 years to do it. I was incredibly intimidated when I arrived at the SHEA headquarters for board orientation. I was the only pediatrician, and nobody knew who I was. In contrast, I knew the names of every person on the board. After several months, I felt comfortable participating in the discussions. As I mentioned earlier, Dr. Louise Dembry was incredibly supportive and helped me gain confidence as a board member.

Those 3 years as the PIDS liaison launched my success as an SHEA member. From 2016 to 2018, I was chosen to be the chair of the CDC/SHEA Outbreak Response Training Program Advisory Panel. In 2018, I became the chair of the SHEA Public Health Task Force. In 2019, I was honored to again serve on the SHEA Board of Trustees, this time as an elected councilor. That same year, I was appointed to serve on the CDC Healthcare Infection Control Practices Advisory Committee (HICPAC).

At this point in my career, I love mentoring early-career physicians. The role of hospital epidemiologist is more challenging than ever, and physicians are often unaware of their value because they are too busy “putting out fires” and attending an endless string of administrative meetings. I enjoy asking mentees to envision what their “ideal job” looks like and hearing what inspires them. I love helping them prepare for their annual reviews or job interviews. Those are exciting moments in everyone’s careers. I frame those moments as opportunities for physicians to highlight their strengths, success, and what they have to offer. We can change the narrative to “You are so lucky to have me here. Let me tell you why.”

I received the SHEA Mentor Scholar Award at IDWeek 2022. It was an incredible honor to be recognized by several colleagues with whom I have worked over the years. I found my own career success because of so many great mentors. Now, I see mentorship and sponsorship as my gifts to “hand down” to younger colleagues. When mentees feel successful, I feel successful too.

## What were some notable barriers in your career and how did you address them?

Finding mentors can be a major barrier for young faculty who are not working in large academic hospital epidemiology programs. This was a major barrier for me when I was getting started. I actively sought mentors outside OHSU. It takes a little bravery to reach out, but it can lead to huge success.

Back in 2010, I was getting frequent phone calls from my local Ronald McDonald House with infection control questions. I thought, “I cannot be the only pediatric hospital epidemiologist getting called by my local Ronald McDonald House!” Eventually, I had an idea to create an infection control guideline for Ronald McDonald Houses. I met a few other pediatric ID SHEA members who were interested in collaborating with me. The problem was that I had no idea how to write a guideline—I did not know where to start. I did not have any local mentors. I did know that I wanted my guideline to become a national resource, like the Cystic Fibrosis Foundation infection control guideline. So, I cold-called Dr. Jane Siegel at her office. She was a past member of Healthcare Infection Control Practices Advisory Committee (HICPAC) and a lead author on the CF Foundation guideline. She loved my idea, and she mentored me throughout the entire process. The final product was the SHEA Guideline, *Infection Prevention and Control in Residential Facilities for Pediatric Patients and Their Families*. I was the first author of the guideline, and Jane was the senior author. We ended up publishing several papers together.

## What career advice would you share with young professionals in epidemiology, public health, or stewardship?

I currently serve as the curriculum director for the CDC-funded “Leaders in Epidemiology, Antimicrobial Stewardship and Public Health (LEAP)” fellowship. The purpose of the LEAP fellowship is to support the development of ID physicians interested in building a collaborative career between clinical healthcare and public health. We train early-career ID physicians how to become bridges that connect hospitals with public health. The LEAP fellowship is a wonderful way for ID physicians to expand their career opportunities.

There will always be new HAI threats, including multidrug-resistant organisms (MDROs), device-associated infections, and novel pathogens. The priorities of our public health HAI program colleagues are the same as hospital IPC programs: to reduce HAIs and antimicrobial resistance. ID physicians must take advantage of these overlapping goals. There are many job opportunities, including direct contract work or contracts through academic institutions, to support faculty salary.

The most important career advice I can give is this: Nobody says, “I wish I worked more” when laying on their deathbed. Yes, we need to work hard, and career success is not simply handed over. But I see many colleagues who work too much. Finding a healthy work–life balance is an important piece of reaching “success.” Our relationships outside work should not suffer. It is just not worth it. Also, one more piece of advice: Proceed until apprehended!

## What books or essays most influenced you (can be outside science or medicine)?

Right after I resigned from my hospital epidemiologist role, I attended a medical conference. At the Portland airport, I bought a book with this intriguing title: *The $100 Startup: Reinvent the Way You Make a Living, Do What You Love, and Create a New Future.* It is a book about entrepreneurship. I read that entire book in 1 day and scribbled many pages of ideas on a note pad. I missed several sessions of the conference because I was so excited. That book helped me realize that our expertise in hospital epidemiology has a variety of applications. It inspired me to use my imagination and to think outside the box, career-wise.

Through my infection prevention consultancy, I have worked with medical device inventors, start-up companies, nonprofit organizations, and the National Basketball Association (NBA). I am the ID consultant for the Trail Blazers, Portland’s NBA team. I have given infection prevention and COVID-19 vaccine lectures to the players and coaches. I led their COVID-19 contact-tracing team. It is amazing to use my hospital epidemiology and infection prevention skills in so many ways. I wouldn’t change anything about my career. I love it.

